# Treatment of clinically severe bovine mastitis – a scoping review

**DOI:** 10.3389/fvets.2024.1286461

**Published:** 2024-01-19

**Authors:** Jensine Wilm, Line Svennesen, Carsten Kirkeby, Volker Krömker

**Affiliations:** Department of Veterinary and Animal Sciences, University of Copenhagen, Copenhagen, Denmark

**Keywords:** antibiotic therapy, supportive therapy, treatment protocol, intramammary infection, clinical mastitis

## Abstract

Mastitis is a major health problem for bovines and can be categorized as non-severe or severe, based on clinical symptoms. A severe case of clinical mastitis is usually defined by the cow being affected systemically. It is important to consider how to handle severe cases because these cases can be fatal and cause high production losses. However, there are generally few detailed treatment guidelines. By conducting a scoping review on the topic, we aimed to synthesize the information that is available on treatment and outcomes, as reported from clinical trials and observational studies. This was facilitated by following the PRISMA-guidelines with a stepwise systematic screening of scientific literature on the subject, retrieved via Pubmed and Web of Science, using pre-defined selection criteria. The results yielded a total of 14 reports of treatment and outcomes in cases of naturally occurring severe clinical mastitis. Cross-trial comparison was difficult due to the different exclusion criteria and outcome definitions. Many studies focused on cases caused by gram-negative bacteria treated with intensive antibiotic protocols, often containing antibiotics that are categorized as critical for human health. Few focused on severe cases caused by gram-positive bacteria or on the relative use of non-antibiotic treatment. In general, only a small number of statistically significant differences were found in trials comparing different treatment protocols, with no obvious trends across trials. Our findings emphasize the need for more research into the treatment efficacy of antibiotic and non-antibiotic options for clinically severe mastitis. Furthermore, consideration of how trial conditions relate to the practical circumstances in a field setting could improve the applicability of reported results. This could help to provide practitioners with the information needed to make evidence-based treatment decisions in cases of clinically severe mastitis.

## Introduction

Mastitis is an inflammatory reaction in the udder tissue, often caused by infectious bacteria, and is considered one of the most important diseases in bovines that has been studied intensively for more than a century (1). Treatment mostly relies on the administration of antibiotics, but therapeutic approaches are continuously evolving (2). Recently, a growing concern about the development of antimicrobial resistance has reinforced the incentive to use antibiotics prudently and to strengthen evidence-based decision making (3). Although there is a broad range of literature available on the subject of mastitis treatment, some topics (e.g., the treatment of severe clinical mastitis cases during lactation) are less reported. Review studies that synthesize evidence on best treatment practice often focus on non-severe cases (4, 5), leaving severe cases to be addressed empirically by field veterinarians without being able to rely on much scientific evidence. Severe clinical mastitis cases can be defined using different criteria, but the usual common denominator is that the cow is systemically affected (6), which can be recognized by clinical signs such as lethargy, anorexia, hyper-or hypothermia or recumbency. The predominance of coliform infections has often been reported in severe mastitis cases (7, 8), leading to a common assumption of a direct link between severe mastitis and gram-negative bacteria. It has since been shown that severe cases are also likely to occur with gram-positive bacterial infections (9), which challenges this assumption. Furthermore, systemically affected cows with severe mastitis have been linked to a high likelihood of bacteremia, which has also been disputed (10, 11). Ideally, all treatment decisions should be based on the full clinical picture as well as paraclinical information, such as the results from milk sample analysis. This would facilitate the targeting of antibiotic treatment toward the pathogen and utilization of knowledge on the highly variable susceptibility and spontaneous cure rates (5). However, severe cases have a high case fatality rate and are considered medical emergencies, where the cow’s survival is the primary concern (12). This sense of urgency means that a delay in treatment due to waiting for laboratory procedures is not considered appropriate. The serious consequences that can result from clinically severe cases of mastitis also affect the way that trials are conducted. Many trials on clinically severe cases of mastitis report the results of induced experimental infections in a controlled environment (13–15). While these trials can inform on aspects of specific infections, they cannot represent the variety of naturally occurring cases present in the field. Severe mastitis treatment practice varies across countries and has been reported to be based on the use of systemic broad-spectrum antibiotics, but also to rely mainly on supportive therapy (16). These reports are approximately in line with previous treatment recommendations for severe coliform mastitis, which proposed the use of systemic fluoroquinolones and cephalosporines to combat associated bacteremia (17). The uncritical use of broad-spectrum antibiotics like fluoroquinolones and high-generation cephalosporins is problematic because they are classified as being critically important in human medicine (18). Furthermore, evidence on the benefits of systemic treatment with, e.g., enrofloxacin is ambiguous, even when considering only *Escherichia coli* mastitis in an experimental setting (19, 20). Furthermore, even though fluoroquinolones are regarded as advantageously effective in veterinary use, a considerably long list of adverse effects has been identified, including a long half-life in the environment (21). For mastitis in general, the relative value of different supportive measures is not clear (22). Hence, it appears that mapping the current situation in the field with a thorough search for studies reporting on treatment and outcomes for naturally occurring severe clinical mastitis could be a valuable first step in creating a better basis for understanding the current practice and improving treatment recommendations. The objective of this scoping review was to assess the extent of information available on the topic of treatment for severe clinical cases of bovine mastitis under field conditions in order to provide an overview for veterinary practitioners and to highlight the knowledge gaps that should be addressed in future studies.

## Materials and methods

This scoping review was conducted according to the PRISMA-ScR principles (23) and the methodological guidance published by the Joanna Briggs Institute (24). A protocol was written in advance and can be provided by the first author on request.

### Systematic search and study selection

Three reviewers with experience in bovine mastitis research and search methodology were involved in the search and the selection process (JW, LS and VK). A literature search was performed on two online platforms, PubMed and Web of Science, on 10th May 2023. The search string contained the following elements: (cow OR bovine) AND (severe OR severity) AND (mastitis) AND (treatment) for all fields. No restrictions on the date of publication were applied. The yielded results were uploaded to the reference management program ‘Endnote’ (25), where automatic deduplication was conducted and followed by a manual check-up. Abstract screening was performed by one reviewer (JW). Reports had to be available in either English, Danish or German. All types of study design were of interest, but only studies published in peer-reviewed journals were included. Furthermore, case reports of single animals were not considered to be extensive enough to qualify for inclusion. All reports with abstracts indicative of a first-hand report of a study describing the treatment of severe bovine mastitis continued to full-text screening, which was performed independently in duplicate by JW and LS. In order to refine the yielded results and meet the objective, the following criteria were added at this stage:

- The reported treatment or intervention should be applied at the onset of clinical symptoms, excluding vaccine studies and second-line treatment studies.
- The report should be on naturally occurring cases, thereby excluding experimentally induced infections because these were not considered representative of field conditions.
- There should be at least one outcome described for the severe cases in particular, meaning it should be possible to distinguish them from non-severe cases. If a large proportion of the studied sample consisted of severe cases, it was also accepted that the outcome was reported for the entire sample.

As ‘severe’ mastitis can be classified based on different criteria, the reviewers emphasized that reports of cases regarded as severe should mention the occurrence of at least one clinical sign of systemic affection (such as fever), in addition to signs of inflammation in the udder. The results of the full-text screening were compared between the two independent reviewers in order to evaluate consensus about final inclusion. The third reviewer (VK) was consulted in cases of doubt. In addition, references from the reports deemed eligible were screened for relevant reports, and these were evaluated for inclusion in this review using the same principles as described above.

### Data extraction and synthesis

Data were extracted from the eligible reports by JW and LS by means of a pre-defined list of items of interest for the objective of this review. The list of items included descriptive parameters on study characteristics, the study population, the conducted treatment, the reported outcome and reasons for inclusion and exclusion of cases within the studies. In terms of presenting the extracted results, the authors intended to keep the presented information as close to the original as possible. However, for some of the items, it was necessary to perform calculations of raw data given in the reports (e.g., the percentage of studies on severe mastitis within a given study sample) and/or to summarize information into a standardized format to create a comprehensible overview (e.g., pathogen information, study purpose, treatment and outcome descriptions, as well as exclusion criteria). Only a selection of the reported outcomes was extracted due to the large variation among studies and because it was not within the objective of this scoping review to perform a meta-analysis. The selection focused on the main outcomes stated by the study authors, relating to the cow-level consequences of a given intervention (e.g., survival or cure).

## Results

**Figure 1:**
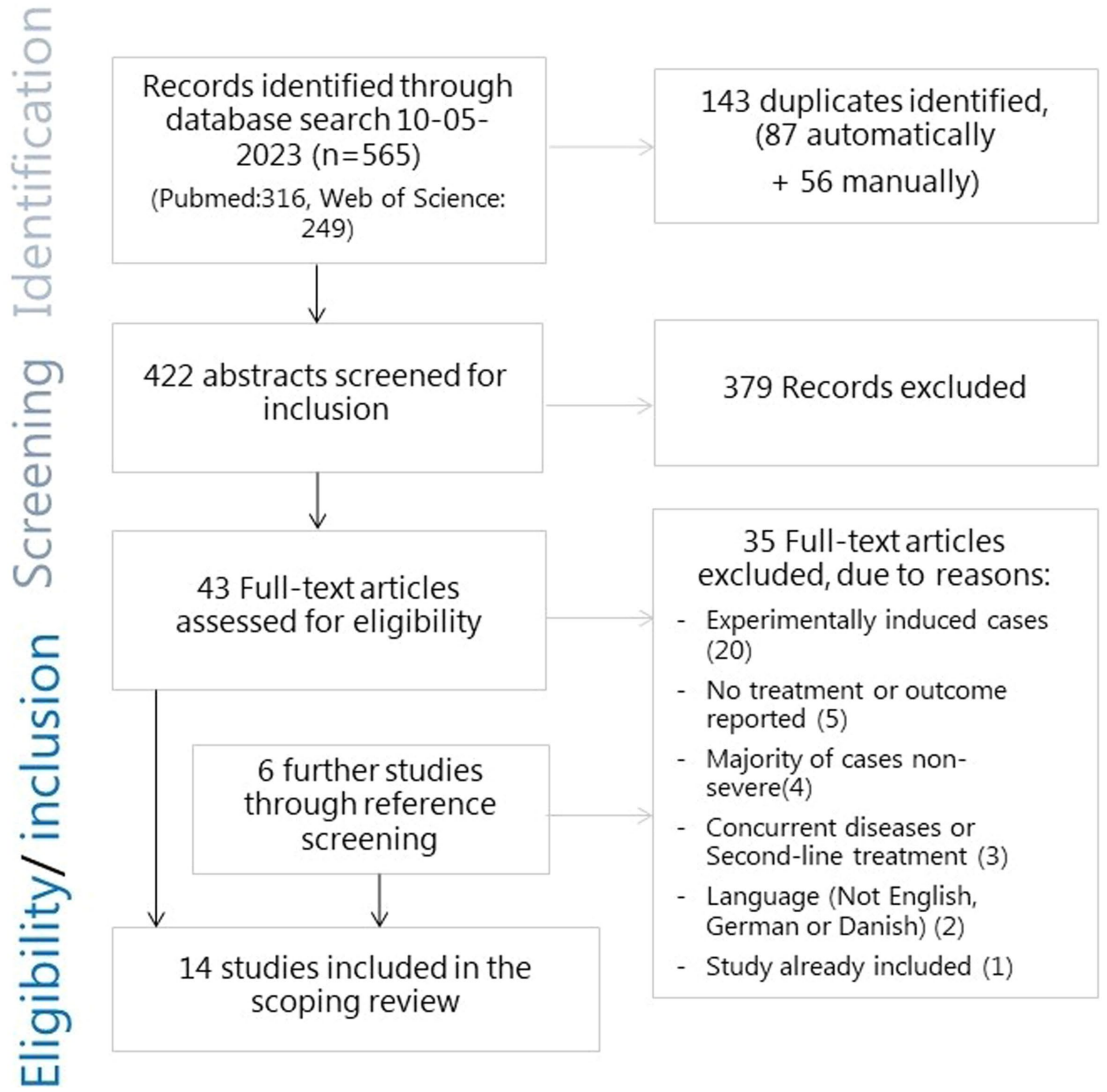
Flowchart of the search and selection process for literature for the scoping review of severe mastitis treatment.

The search for literature yielded 422 abstracts after 143 duplicate reports were removed. Of these, 379 reports were excluded after the abstract-screening stage, while 43 proceeded to the full-text screening. An additional six reports were identified through reference screening. Severe cases were sometimes initially described using different phrasing in the abstract, such as ‘toxic mastitis’ or ‘acute coliform mastitis’. In case of doubt, reports automatically proceeded to the full-text screening. Thirty-five reports were excluded at the full-text-screening stage due to the following reasons: not being a study of naturally occurring infections (*n* = 20); not including information on treatment (*n* = 3) or outcome (*n* = 2); cases not being severe in the sense of the cows being systemically affected (*n* = 2) or only a minor fraction of the sampled cases being severe; that it was not possible to separate the outcome from non-severe cases (*n* = 2); cases with concurrent diseases (*n* = 1) or that were treated prior to study enrollment (*n* = 2); study written in Dutch (*n* = 2); reporting on the same study (*n* = 1). This led to a total of 14 reports of studies in which naturally occurring severe cases of clinical mastitis were treated and an outcome was reported, thus making them eligible for inclusion in this review. The selection process is shown in Figure 1.

### Study characteristics

An overview of the studies (numbered) and their type and purpose is given in Table 1. Out of the 14 yielded reports, nine were clinical trials and eight of these used predefined randomization procedures for treatment protocols, while one left the choice of treatment up to the veterinarian (11). Two of the clinical trials used a degree of blinding. In one of these, the veterinarian performing the clinical examinations post-treatment was blinded to the received treatment (4) and in the other, the farmers giving the treatments were provided with a placebo (a saline solution) with the same appearance as the antibiotic substance used (12). Three of the clinical trials compared the efficacy of several antibiotic treatment protocols (4, 7, 11), three compared an antibiotic protocol to a control group (2, 12, 13) and three studies compared supportive treatments or interventions (5, 8, 9). The remaining five studies were three descriptive case reports (1, 6, 10) and two case–control studies comparing pharmacological properties of administered drugs in severe mastitis cases compared to healthy cows (3, 14).

**Table 1:** Overview of study characteristics and sample population characteristics from the 14 yielded reports of severe mastitis treatment.

	Author (Year)	Study type	Summarized study purpose	Severe cases	Total cases	% severe cases^1^	Pathogen information for severe cases^1^
1	Bättig et al. (26)	CR	Descriptive study of mastitis caused by *Nocardia* infections	6	7	86%	All *Nocardia*, 1 co-infection with *E.coli*
2	Erskine et al. (27)	ORT	Efficacy study comparing a systemic antibiotic treatment protocol to a control group receiving intramammary treatment for severe mastitis	104	–	100%	42% *E.coli*, 12% *Klebsiella* spp., 20% No growth, 14% *Streptococci* spp., 9% *Staphylococci* spp., 4% Others
3	Gorden et al. (28)	CCS	Comparison of plasma pharmacokinetics in ceftiofur-treated severe mastitis cases vs. healthy cows	8	–	100%	63% *E.coli* and 38% *Klebsiella* spp.
4	Grandemange et al. (29)	BRT	Product registration study investigating non-inferiority in the efficacy and safety of two antibiotic treatment protocols for *E.coli* mastitis	354	–	100%	51% *E.coli*, 22% *Streptococci* spp., 12% No growth, 5% Others, 5% Mixed infections, 3% *Staph. Aureus*., 3% *Klebsiella* spp.
5	Green et al. (30)	ORT	Efficacy study investigating the addition of three supportive treatments to antibiotic treatment protocols for severe mastitis	54	–	100%	52% Coliforms, 20% Contaminated, 13% No growth, 11% No results available, 4% *Strep. uberis*
6	Hagiwara et al. (31)	CR	Retrospective comparison of prognostic factors in survivors vs. non-survivors of *E.coli* mastitis	24	–	100%	All *E.coli*
7	Jarp et al. (32)	ORT	Efficacy study comparing three different antibiotic protocols for mastitis caused by penicillin-sensitive agents	146	439	33%	Not available for severe cases only, but for entire trial: 63.6% *Staphylococcus aureus*, 24.1% *Streptococci* spp. 12.3% Other penicillin-sensitive bacteria
8	Krömker et al. (33)	BRT	Efficacy study investigating the addition of NSAIDs to antibiotic treatment protocols for severe mastitis	69	–	100%	26% *Streptococci* spp., 23% No growth, 17% *Klebsiella* spp., 9% *E.coli*, 20% Others, 4% Contaminated
9	Krömker et al. (34)	ORT	Efficacy study investigating the effect of increased milking frequency in conjunction with antibiotic treatment for clinical mastitis	12	107	11%	Not available for severe cases only, but for entire trial: 36% No growth, 33% *Streptococci* spp., 12% *E.coli*/coliforms, 12% Others, 5% *Staph. aureu*s, 3% Mixed infections
10	Oliveira et al. (35)	CR	Descriptive study of clinical mastitis treatments in Wisconsin dairy herds	89	589	15%	Not available for severe cases only, but for entire sample: 29% *E.coli*, 9% *Klebsiella* spp., 16% *Streptococci* spp., 8% Others, 4% *Staph. aureus*, 35% No growth
11	Perner et al. (36)	OT	Efficacy study comparing three antibiotic treatment protocols for clinical mastitis	37	243	35%	Not specified, but mainly *Staphylococci*, *Streptococci* and some *Enterobacteriaceae* in entire trial
12	Persson et al. (37)	BRT	Efficacy study comparing an antibiotic treatment protocol to a placebo for *E.coli* mastitis	52	56	93%	All *E.coli*
13	Suojala et al. (38)	ORT	Efficacy study comparing an antibiotic treatment protocol to NSAID treatment for *E.coli* mastitis	105	132	80%	All *E.coli*
14	Walker et al. (39)	CCS	Descriptive study of the effect of flunixin meglumine on biomarkers and oxidative stress in *E.coli* mastitis	8	–	100%	All *E.coli*

^1^Calculation of % and grouping of pathogens were performed in some cases to present studies concisely.

CR, case report ORT, open randomized trial, CCS, case–control study, BRT, blinded randomized trial, OT, open trial.

### Study population

The study populations are described in Table 1. The number of severe mastitis cases enrolled in the 14 studies varied from 6 to 354 cases. In three of the clinical trial studies (7, 9, 11) and one case report (10), only a minor proportion of the full study population were classified as severe. For these four studies, the outcome was specified but pathogen information was not stratified for case severity. Two trials stood out by having a majority of gram-positive bacteria identified as causative pathogens (7, 11). For the remaining 10 studies, the main proportion of enrolled cases were classified as severe (80–100%). Of these, four studies included *E.coli*-positive cases only (6, 12, 13, 14), one case report study described *Nocardia*-positive cases alone (1), while the remaining five reported a range of different causative pathogens (2, 3, 4, 5, 8).

### Reported treatments

Treatments are summarized separately for: the three trials comparing several antibiotic protocols (Table 2); the three trials comparing an antibiotic protocol to a control group (Table 3); the three trials comparing supportive treatments or interventions (Table 4); the three observational case reports and two case–control studies (Table 5). Of the trials comparing several antibiotic protocols, virtually all had at least one group treated with combined parenteral and intramammary antibiotics, while the other groups were typically treated with either intramammary or parenteral antibiotics for different durations. For the trials comparing one antibiotic protocol to a control group, the control group also received either intramammary pirlimycin (2), intramammary penicillin G until a diagnosis of *E.coli* was confirmed (13) or optional benzyl penicillin if the veterinarian considered it necessary (12). The rationale for using these intramammary treatments in addition to the investigated treatment protocol of these studies was the assumed ineffectiveness against the targeted pathogen (*E.coli*). Likewise, a study comparing two types of fluoroquinolone-based parenteral antibiotic protocols (4) also administered intramammary oxacillin for all cows. In the three trials investigating supportive treatments or interventions, all cows received combined parenteral and intramammary antibiotics. The investigative focus within the trials was on a diverse range of different types of antibiotics—from fluoroquinolones (4, 8, 9, 12, 13) or 3rd-and 4th-generation cephalosporines (2,8, 9) to penicillins (benzyl-penicillin and amoxicillin) alone and in combination with aminoglycosides or beta-lactamase inhibitors (7, 11) and tetracyclines (5). Likewise, a wide range of applied antibiotics were reported within the five observational studies (Table 4). The route of administration also varied between and within the observational studies. While some cases received combined parenteral and intramammary treatment (1, 6, 10), others were treated with parenteral (3, 10, 14) or intramammary antibiotics only (10). Supportive treatments were reported as optional or conducted for all cases in all but two (1, 11) of the 14 studies. The supportive measures described were particularly diverse, including different combinations of oxytocin, fluids, calcium, glucose, heparin, anti-inflammatory drugs and frequent milking. The three trials that revolved around comparing supportive measures focused on the administration of IV isotonic fluids (5) and/or non-steroidal anti-inflammatory treatment (5, 8) or increased milking frequency (9).

**Table 2:** Overview of treatment protocols and results of three clinical trials comparing several antibiotic protocols for severe mastitis cases.

Report	Treatment protocol I	Treatment protocol II	Treatment protocol III	Other treatment	Extracted outcome	Results
4	G1: 1× 10 mg/kg IM marbofloxacin	G2: 1× 6 mg/kg SC danofloxacin	NA	All cows: 3 days IMM oxacillin, Cows with anorexia: 500 mL hypertonic glucose Optional: fluid therapy	BC on days 15 & 27 ‘Success’ on day 15, defined by either CC (absence of clinical signs) or clear improvement (absence of general clinical signs but tolerance of local signs)	Per protocol results for *E.coli*-positive sub-sample (*n* = 148): non-inferiority between two treatments measured by ‘success’ on day 15 (G1: 86%, G2: 82%) and BC on days 15 & 27 (G1: 79% G2: 70%) Intention-to-treat results: all assessable enrolled cows (*n* = 354 minus the unknown number of excluded cases) ‘success’ on day 7 is claimed similar to per-protocol results
7	G1: 1x combined IM benzyl-penicillin-procaine & dihydrostreptomycin (10^6^ IU) and 1x IMM benzyl-penicillin-procaine (200 mg) & dihydrostreptomycin (250 mg) at onset, followed by 3x IMM benzyl-penicillin-procaine and dihydrostreptomycin at 24 h int.	G2: 3x IM benzyl-penicillin-procaine (10^6^ IU) at 24 h int.	G3: 5x IM benzyl-penicillin-procaine (10^6^ IU) at 24 h int.	All cows: Oxytocin IV, stripping every 2 h	Overall cure defined by BC & CytC at quarter level on day 24–26 (number of quarters unknown)	For severe cases: G1: 38.5%, G2: 35.1%, G3: 42.7%, Stratified for *Staph. aureus*: G1: 31.9%, G2: 24.5%, G3: 32.1%, no significant difference between groups
11	G1: 3x IMM amoxicillin (200 mg) & clavulanic acid (50 mg) and prednisolone (10 mg) at 12 h int.	G2: Combined: 1x IM amoxicillin (7 mg/kg) and clavulanic acid (1.75 mg/kg) at onset and 3x IMM amoxicillin (200 mg) & clavulanic acid (50 mg) and prednisolone (10 mg) at 12 h int.	G3: 2x IM amoxicillin (7 mg/kg) and clavulanic acid (1.75 mg/kg) at 24 h int.	NA	CC & BC, separate and together, at quarter level on days 14 & 21 (not all reported)	For severe cases: G1: (2 quarters) No information G2: (38 quarters) BC day 14/21: ~74%/66%, BC & CC day 21: 26% (stratified for *Staphylococci*: 7% and *Streptococci*: 33%) G3 (7 quarters): BC & CC day 21: 29%

int., interval; IM, intramuscular; IMM, intramammary; SC, subcutaneous; IV, intravenous; BC, bacteriological cure; CC, clinical cure; CytC, cytological cure.

**Table 3:** Overview of treatment protocols and results of three clinical trials comparing an antibiotic protocol to a control group for severe mastitis cases.

Report	Investigated protocol	Control group	Other treatment	Extracted outcome	Results
2	G1: 5x IM 2.2 mg/kg IM ceftiofur at 24 h int.	G2: No PE antibiotics	All cows: 1–3x IMM pirlimycin at 24 h int. and typically: hypertonic saline IV, 500 mL calcium borogluconate SC, 20 mg isoflupredone acetate IM or 1.1 mg/kg flunixin meglumine IV, oral fluids. Optional: other drugs or vitamins	Survival (culled or dead within 30 days)	G1: 8%, G2: 19% dead, no significant difference between groups. Stratified for coliforms: G1: 14% G2: 37% dead, significant difference in favor of ceftiofur-treated group (*p* < 0.05)
12	G1: 3× 2.5 mg/kg IM enrofloxacin at 24 h int.	G2: 3x IM saline solution (placebo) at 24 h int.	All cows: benzylpenicillin if veterinarians considered it necessary, 1x IM 0.5 mg/kg meloxicam. Optional: frequent milking, fluids, calcium and oxytocin	Survival, CC day 3, BC day 22–28	For severe and non-severe cases: G1: 6/34 dead, CC: 21%, BC: 88%, G2: 3/22 dead, CC: 11%, BC: 84%, no significant difference between groups
13	G1: 2× 5 mg/kg enrofloxacin, first dose IV, second dose SC at 24 h int.	G2: No PE antibiotics	All cows: IMM penicillin G (600 mg) until *E.coli* diagnosis was confirmed, 3 mg/kg and ketoprofen IV or IM or 4 mg/kg *per os* for 1–3 days. Optional: fluids and frequent milking	Survival for 3 weeks, CC and BC days 2 & 21, from analysis day 2: 34 missing and for day 21: 52 missing cases	For severe and non-severe cases: G1: 3/64 dead, CC day 2: 8%, CC day 21: 47%, BC day 21: 91% G2: 5/68 dead, CC day 2: 20%, CC day 21: 57%, BC day 21: 87%, no significant difference between groups, except for day 2 (BC in favor of enrofloxacin, while CC in favor of non-treated group, not further specified)

int., interval; IM, intramuscular; IMM, intramammary; SC, subcutaneous; IV, intravenous; PE, parenteral; BC, bacteriological cure; CC, clinical cure; CytC, cytological cure.

**Table 4:** Overview of treatment protocols and results of three clinical trials comparing supportive treatments or interventions for severe mastitis cases.

Report	Treatment protocol I	Treatment protocol II	Treatment protocol III	Other treatment	Extracted outcome	Results
5	G1: Protocols II and III combined	G2: 30 L isotonic fluid IV at onset, 15 L ~ 24 h later (unless complete recovery)	G3: 2x flunixin 1,000 mg IV at onset and ~ 24 h later	All cows: combined oxytetracycline (2,000 mg) (2x IV, followed by 3x IM) and 5x IMM chlortetracycline (420 mg) + hydrocortisone (2 mg) for a total of 5 days. 1x calcium borogluconate IV 1x oxytocin (80 IU) followed by stripping at onset. Optional: frequent milking	Survival	G1: 8/18, G2: 8/18 G3:9/18 dead, no significant difference between groups
8	G1: Standard farm protocols without NSAIDs	G2: standard farm protocols with 1x SC carprofen (1.4 mg) at onset	NA	Cows at farm 1 & 2: combined 2x IM cefquinome at 24 h int. and 3x IMM cefquinome at three milkings. Cows at farm 3: combined 3x IM marbofloxacin daily and 3x IMM benzylpenicillin at 24 h int. + 35 L oral fluids	Survival, BC and CytC 14 & 21 days after withdrawal, 16 no-growth cases were only analyzed for survival	G1: 5/69 dead, 61% BC, 25% CytC G2: 3/69 dead, 76% BC, 40% CytC, no significant difference between groups
9	G1: milked twice daily	G2: milked four times daily	NA	All cows with severe mastitis: Combined SC enrofloxacin, IMM cefquinome and IV flunixin meglumine (1.1 mg/kg)	CC, BC and CytC 14 & 21 days after withdrawal	For severe cases, G1: CC:2/4, BC: 3/4, CytC:1/4, G2: CC: 5/8, BC: 6/8, CytC: 4/8, no significant difference between groups

int., interval; IM, intramuscular; IMM, intramammary; SC, subcutaneous; IV, intravenous; BC, bacteriological cure; CC, clinical cure; CytC, cytological cure.

**Table 5:** Overview of treatments and results for severe mastitis treatments in the three case reports and two case–control studies.

Report	Description of applied treatment	Extracted outcome	Results
1	Intensive combined antibiotic treatment for several days in all cases	Survival	All six severely affected cows were culled within 14 days
3	All cows: 5× 2.2 mg/kg IM ceftiofur at 24 h int. 8/8 disease-group cows 20–40 L oral fluids for an average of 3.9 days, 7/8 disease-group cows received 2.2 mg/kg of flunixin meglumine IV for an average of 2.3 days, 5/8 disease-group cows received 3–5 mL/kg hypertonic saline IV for an average of 1.2 days	Survival	6/8 cows culled (5 immediately after 10-day trial, 1 within 60 days of follow-up)
6	All cows: IM kanamycin sulfate (4,000–6,000 mg/cow/day), IMM kanamycin sulfate (300 mg/cow/day) & penicillin-G-procaine (300,000 U/cow/day), hypertonic saline IV (2,000 mL/cow/day) Optional: 1,000 U of heparin sodium IV (25–50 mL/cow/day), physiological saline solution IV (2,000–8,000 mL/cow/day) and 5% glucose IV (2,000–5,000 mL/cow/day)	Survival	7/24 cows died or were euthanized within 8 days
10	Of 89 severe cases: 13.5% treated with 2 concurrent PE antibiotics, 20.2% treated with an IMM antibiotic (ceftiofur or cefapirin), 43.8% treated with a single IMM antibiotic dose combined with a PE antibiotic (ampicillin, ceftiofur, oxytetracycline, sulfadimethoxine, florfenicol or combined spectinomycin and lincomycin), 22.5% received an additional secondary treatment (either IMM, PE or both), 48.3% received supportive therapy, including fluids, calcium, hypertonic saline and anti-inflammatory drugs	Survival for 90 days, Days until CC (outcome was only reported sporadically)	For reported severe cases: 16 received PE sulfadimethoxine: 3 dead, 16 cases treated with IMM ceftiofur: days until CC: 2–6 days, 2 cases treated with IMM cefapirin: all culled
14	All cows: 1× 2.2 mg/kg SC ceftiofur sodium, disease-group cows: 1× 2.2 mg/kg IV flunixin meglumine and oral electrolyte fluids	Survival during study period	4/8 dead

int., interval; IM, intramuscular; IMM, intramammary; SC, subcutaneous; IV, intravenous; PE, parenteral; BC, bacteriological cure; CC, clinical cure; CytC, cytological cure.

### Reported outcomes

The selected extracted outcomes are described next to the treatment protocols in Tables 2–5, while reasons for inclusion and exclusion of cases are described in Table 6. Survival was the most common result, reported in 10 studies (1, 2, 3, 5, 6, 8, 10, 12, 13, 14), while seven reported on cure (clinical, bacteriological and/or cytological) at different time points (4, 7, 8, 9, 11, 12, 13). Survival within the observational studies was generally low, with 19–100% (mean = 64%) dead or culled cows reported despite treatment (1, 3, 6, 10, 14). In contrast, survival within the clinical trials was remarkably high, with four trials reporting not a single case fatality (4, 7, 9, 11), four trials reporting a relatively low range of 4–19% dead cows (2, 8, 12, 23) and only one trial reporting a relatively high case fatality rate of 44–50% dead cows despite treatment (5). The type (s) of cure reported as well as the number of days between treatment and post-treatment measurements varied considerably across studies. Two studies reported individual results for two types of cure (bacteriological and clinical) from two separate post-treatment measurements (11, 13). One study reported two types of cure (clinical cure day 3 and bacteriological cure day 22–28) from two individual post-treatment measurements (12). Three studies reported the combined results of two post-treatment measurements (typically days 14 and 21) for the individual types of cure (4, 8, 9), while two reported combined results of different types of cure (e.g., bacteriological and clinical or cytological cure) from one post-treatment measurement (7, 11). One study presented the results as a combined definition termed ‘success’ (4), covering clinical cure or clinical improvement of systemic signs, while another study reported the days until clinical cure (10). Due to these differences in reporting, the range of outcomes for ‘cure’ was large both between and within studies, making them difficult to synthesize. In general, few within-trial comparisons of treatment groups yielded statistically significant results. In some studies, the number of enrolled cases (presented in Table 1) did not match the number of cases for which the full results were reported (Tables 2–5). Cases were excluded from the analysis due to criteria related to the microbiological results (4, 8), for unstated reasons (10, 11) or for various reasons including missing recordings (13). Some of the clinical trials stated that deviations from the treatment protocol or the need for additional treatment led to exclusion from the analysis (4, 11, 12, 13). Three trials reported cases requiring additional treatment as ‘treatment failures’, but did not exclude them (2, 8, 9), while one trial used pre-treatment analysis results as control values for cows requiring additional treatment (7). This post-enrollment exclusion could reduce the sample size considerably, with some studies fully reporting on only ~40% of the initially presented severe mastitis cases (4, 10). Three of the clinical trials presented results for all enrolled cases as primary results, while partly analyzing a subgroup of causative pathogens (2, 7, 11). Two studies presented outcomes at quarter level (7, 11), while the remaining studies presented outcomes at cow level. One trial (4) presented full results for only the *E.coli*-positive cases as the primary finding (per-protocol analysis), while the full sample (intention-to-treat analysis) was partly analyzed and the results mentioned, although the corresponding data were not shown. In addition, many of the clinical trials applied exclusion criteria prior to enrollment and presentation of the sample (Table 6). The pre-enrollment exclusions were primarily due to a prerequisite of cases being confirmed as *E.coli*-related (12, 13, 14) or due to other criteria related to results of the microbiological analysis (7, 11). Reasons for the pre-enrollment exclusion of cases not related to microbiological results were very diverse. Only one of the clinical trials did not state pre-enrollment criteria for the inclusion of cases (5). Many of the remaining trials shared some of the same inclusion criteria. Among the most frequent criteria were that the cow did not suffer from concurrent diseases, had only one affected quarter, showed no signs of teat trauma and received no other treatment close to enrollment. However, these requirements were not always used, and rarely in the same combinations. The differing inclusion and exclusion criteria used prior to and after enrollment, as well as reported outcomes, indicates a large degree of heterogeneity across clinical trials on severe mastitis treatment in general.

**Table 6:** Stated reasons for inclusion and exclusion of cases prior to enrollment in nine clinical trials of severe mastitis treatment.

Report	Criteria related to microbiological analysis	Other criteria for exclusion
2		>1 affected quarter Concurrent diseases Teat trauma in affected quarter Previously enrolled History of chronic mastitis IMM mastitis treatment in affected quarter within 30 days before enrollment
4	Only microbiologically confirmed *E.coli* cases were fully reported and analyzed (160 cases with other pathogen results excluded, corresponding to 45% of the original sample)	>1 affected quarter Concurrent diseases Teat trauma in affected quarter Received antimicrobials and/or anti-inflammatories (PE or IMM) within 30 days Vaccinated against *E. coli* Cows producing <5 L milk/day Requiring additional treatment
5		None stated
7	Only penicillin-sensitive agents included (44 no-growth cases and 124 cases infected with penicillin-resistant bacteria excluded, corresponding to 26% of original sample)	Teat trauma Previously treated within same lactation >3rd lactation > 6 months from parturition
8		>1 affected quarter Concurrent diseases Teat trauma Previously enrolled Chronic mastitis or antibiotic or anti-inflammatory treatment within previous 14 days Cows with dry quarters
9		Disease within 14 days before trial initiation Cows with repeated clinical mastitis in one quarter
11	Exclusion of cases with contaminated samples (*n* = 13), no growth (*n* = 14), ‘rare’ pathogens (*n* = 7), corresponding to 22% of original sample	Concurrent diseases Antibiotic treatment in the previous 4 weeks
12	Only microbiologically confirmed *E.coli* cases (54 cases with other pathogen results excluded, corresponding to 47% of original sample)	Deviation from treatment protocol
13	Only microbiologically confirmed *E.coli* cases (number of cases with other pathogen results unspecified)	>1 affected quarter Vaccinated against *E.coli* Deviation from treatment protocol

## Discussion

To our knowledge, this is the first attempt to perform a systematic search and to review the scope of clinically severe mastitis treatment under field conditions. Clinical trials on the treatment of severe mastitis varied widely in terms of inclusion criteria, applied treatments and reported outcomes. This indicates considerable differences in the treatment approach for severe mastitis, but unfortunately does not provide an ideal setting for cross-trial comparison. The number of clinical trials on severe, naturally occurring cases of mastitis in a field setting was limited, as expected. This is likely due to ethical concerns about the high case fatality rate and impact on animal welfare, lowering the incentive to apply treatments that would potentially be inferior.

The majority of studies focused on gram-negative agents in the analysis and their treatment protocols typically included systemically administered broad-spectrum antibiotics, such as quinolones or 3rd-and 4th-generation cephalosporines, either alone or accompanied by intramammary treatments. Studies focusing on gram-positive agents generally used penicillins (benzyl-penicillin and amoxicillin), in some cases accompanied by beta-lactamase inhibitors or aminoglycosides. These treatment protocols align to a large extent with the current state of available general treatment recommendations for severe mastitis, supporting parenteral treatment (40). The European medicines agency (EMA) states that negative control field studies of mastitis are usually not acceptable for *E.coli* infections for welfare reasons (41), and it is assumed that they refer to clinically severe cases in this regard. Control groups within the reviewed studies were mostly positive, in the sense that they received similar antibiotics but varied in terms of treatment duration or route of administration. However, there were also trials comparing intensive systemic protocols with broad-spectrum antibiotics intended for *E.coli* or other coliform bacteria to a control group receiving only narrow-spectrum intramammary antibiotics that would not be expected to have any effect on coliforms. These trials were conducted in Scandinavia, where the prudent use of antibiotics has been systematically implemented through legislation over the past decade (42). The Nordic guidelines for mastitis treatment specifically recommend supportive therapy alone as the first choice of treatment for clinical mastitis caused by *E. coli* (43). In recent years, many other European countries have likewise adopted a restrictive use of some critical antibiotics (16) that were typically used in the reported studies. The national context and timeframes are therefore presumably accountable for some of the differences in the trial designs.

Based on these differing designs and a lack of significant differences demonstrated between groups within the trials, it is difficult to argue whether systemically applied antibiotics will improve the chances of the cow recovering following severe mastitis. The most obvious rationale for systemic antibiotic treatment in cases of severe mastitis would be to target bacteria in the blood. While it has previously been reported that there is a high risk of severe mastitis being accompanied by bacteremia [at approximately 32%; (44, 45)], recent studies report levels of 1.4 and 15.5% (10, 11), suggesting that the risk is much lower. In trials with experimentally induced mastitis, a statistically significant enhanced bacteriological clearance in enrofloxacin-treated cows was demonstrated by one study (46) but not another (47). Furthermore, a third study found no reduction in clinical signs, although milk production did improve in treated cows (19). The *in vitro* sensitivity of targeted pathogens toward the given treatment could be responsible for some of the effect on the outcome (48), but this has not been consistently addressed in the studies.

The main reason for excluding studies from this review was that they reported on experimentally induced rather than naturally occurring mastitis. These studies mostly used an infusion of *E.coli* bacteria or corresponding endotoxins. While these studies can contribute valuable information relevant to severe mastitis cases associated with *E.coli* infections, they neglect to address severe cases of different etiology. *E.coli* and other coliform bacteria may be the predominant cause of severe mastitis, but it is important to consider that other agents can also present the same clinical picture of severe mastitis (49). A diverse etiology in severe mastitis cases was also shown in the studies in this review. In a field setting, the clinical presentation has a great influence on decision-making at treatment initiation (50). Unfortunately, indications like inspection of the visual appearance of mastitic milk are not enough to draw conclusions about the causative pathogen (7). This creates a dilemma when it comes to designing representative studies on the best treatment practice for severe mastitis, where swift treatment initiation is necessary. Several of the reviewed studies chose to exclude cases based on etiology retrospectively. This practice is logical when aiming to prove the efficacy of a given therapy toward a targeted pathogen, as the EMA recommends it (41). However, similar to the studies on experimentally induced mastitis, it has the disadvantage that results are not representative of a field setting. Interestingly, these studies often investigated a systemic antibiotic treatment aimed at suspected gram-negative agents, while simultaneously applying local antibiotics targeting gram-positive agents. Simultaneously applying several treatments that target different causative bacteria may be a way of ensuring the best chances of recovery, but at the same time it goes against the aim of using antibiotics prudently. It is also important to consider the pharmacological properties of the applied antibiotics, in particular the effect that a severe clinical presentation might have on the distribution (51).

The observational studies we reviewed employed a very large variety of applied treatments. While some reported several days of intensive combined antibiotic therapy, other cases only received a single dose administered either by systemic or intramammary route. Supportive therapy was not reported much in detail, but it appears that the use of non-steroidal anti-inflammatory drugs (NSAIDs), hypertonic saline and other fluid therapy is fairly common. Supportive treatment was also extremely diverse across the studied trials and only three of the reviewed studies focused on investigating the benefits of different supportive measures in the context of severe mastitis. Current treatment recommendations for severe mastitis encourage the use of supportive treatment, including fluids (52) and anti-inflammatory drugs (17) alongside parenteral antibiotics. A positive effect of NSAIDs has been demonstrated in a field trial on mastitis caused by coliform bacteria (48). Fluid therapy in cattle is a complex theme with many aspects and options to consider (53), and evidence for its applicability in severe mastitis cases might be easier to find in a general context. Intravenous administration of isotonic fluids in larger volumes, or smaller volumes of hypertonic fluids at ambulatory visits have been suggested alongside potential supplementation with calcium, potassium and glucose (54), based primarily on studies of cows with endotoxin-induced mastitis.

The outcomes reported ranged from different production parameters to cure rates and survival. Survival was the most commonly reported outcome, although the follow-up period varied. Interestingly, many of the trials reported that no cows within the studied population died. In contrast, the case fatality rate was often high in the observational studies. A cross-sectional study on severe mastitis cases described a case fatality rate of 27%, despite treatment, over a 60-day follow-up period, including cows that had been euthanized (12). Another study focusing on coliform cases reported 19% case fatality in treated severe cases (55). There could be several explanations for the large variation in survival rates across the reviewed studies. For example, even though severe cases constitute a distinct category, this still can cover a wide range of clinical presentations. In addition, the trials only report on the selection of patients that meet the inclusion criteria, which could limit the extent to which very serious cases of severe clinical mastitis are enrolled—for example, excluding cows with more than one affected quarter or cases needing additional treatment would add a potential bias to the interpretability of results.

Cure types were reported in different combinations and using different definitions, making it difficult to compare outcomes. Likewise, the reasons for exclusion prior to enrollment and prior to the analysis determined very different pre-conditions for the investigated groups across trials. The EMA guidelines suggest that the primary parameter of efficacy evaluation in mastitis treatment is bacteriological status, followed by clinical status as the secondary parameter. Furthermore, they suggest two milk samples are collected between 14 and 28 days post-treatment (41). An important issue to address in future studies on severe mastitis treatment is whether bacteriological cure is actually the most relevant outcome, or if production parameters, survival and clinical cure should be emphasized. Other outcome categories, like milk yield development or remaining in the herd for a longer period, were also considered in some of the studies, but are not covered in this synthesis. Furthermore, we did not go into detail with potential differences between the reported laboratory procedures or specific details of cure definitions at study level. A recent review on the reported outcomes of antibiotic efficacy studies for bovine mastitis demonstrated that definitions of reported cure rates vary widely across studies and that there is no consistent association between bacteriological and clinical cure (56). Hence, the suggested effect of a given treatment might be greatly influenced by choices made about the definition of the outcome.

There may be some limitations to the search methodology, including a potential oversight of reports using different terminology for the topic of interest or unpublished work. However, we aimed to have a stringent and transparent process in order to produce trustworthy and reproducible results (24). While a scoping review is not designed to produce a meta-analysis, the results of this investigation can provide an overview and a baseline for further development of severe mastitis treatment studies and practice.

## Conclusion

Few studies on the treatment of naturally occurring severe bovine mastitis are available in the published scientific literature reviewed here. The studies that do exist are characterized by high heterogeneity in the sample population, applied treatments and reported outcomes. Not many statistically significant differences between treatments within the clinical trials were reported. In the light of increased focus on antibiotic resistance, the efficacy of critical agents is an important issue to address in future studies of clinically severe mastitis treatment. Additionally, the potential benefits of supportive therapies should be explored further. Several trials focused only on severe cases caused by coliform bacteria, while the causative pathogen will often be unknown at treatment initiation. The uncertainty of pathogen-status should be considered and included in future study designs. One way to address the diverse etiology in a field setting in future studies could be to differentiate results, like some studies did, presenting intention-to-treat results on all enrolled cases, as well as per-protocol results stratified for the targeted pathogen. Furthermore, the uncertainty about the true prevalence of bacteremia should also be considered. Updated guidelines supporting the alignment of enrolment criteria and reported outcome definitions could improve our ability to synthesize evidence across studies in the future.

## Author contributions

JW: Conceptualization, Data curation, Formal analysis, Investigation, Methodology, Project administration, Validation, Visualization, Writing – original draft, Writing – review & editing. LS: Data curation, Formal analysis, Investigation, Methodology, Validation, Visualization, Writing – review & editing. CK: Conceptualization, Project administration, Supervision, Writing – review & editing. VK: Supervision, Validation, Writing – review & editing.
